# ‘Flow’ Transcranial Direct Current Stimulation (tDCS) Device and Behaviour Therapy Training Software Used at Home for Community Mental Health Team (CMHT) Patients With Symptoms of Depression

**DOI:** 10.1192/bjo.2024.156

**Published:** 2024-08-01

**Authors:** Chris Griffiths, William McIlhiney, Alex O'Neill-Kerr, Kate Walker, Simon Stringer

**Affiliations:** Northamptonshire Healthcare NHS Foundation Trust, Northampton, United Kingdom

## Abstract

**Aims:**

Flow is a transcranial direct current stimulation (tDCS) treatment for depression without major side effects that patients use at home. Over 30 years of research/clinical use show tDCS is safe (Razza et al., 2020). Flow is CE-marked for treating depression in Europe. Recent NICE briefing published (NICE, 2023). The patient self-administers and remains awake (NICE, 2015), treatment sessions last for about 30 minutes, and are repeated 5 times weekly for three weeks (Flow, 2023). After the initial three-week period, patients self-administer 3 sessions per week for 3 weeks, and then as long as required (Flow, 2023). Meta-analyses of randomised sham-controlled trials (RCT) show tDCS is associated with significant improvements in depressive symptoms and high rates of clinical response and remission relative to placebo sham stimulation (Mutz et al., 2018, 2019; Moffa et al., 2020; Razza et al., 2020). Flow RCT study depression remission rates are 45% (Fu et al., In Press). Flow incorporates an evidence backed healthy lifestyle behaviour training software app, and depression symptom tracking that enables users to monitor their progress/symptoms. Training modules on: ‘Behaviour activation’, ‘Mindfulness’, ‘Exercise for your brain’, ‘An anti-depression diet’, and ‘Therapeutic sleep’. Flow also provides an integrated platform for clinicians to monitor use and depression symptoms.

In a first for the NHS, in a post-marketing informed consent study, NHFT's community mental health team (CMHT) offered Flow to their patients with a diagnosis of depression and evaluated the feasibility and impact.

**Methods:**

Outcome measure data collection from baseline to 6 week follow-up point. Self-report measures used were depression: Personal Health Questionnaire (PHQ-9) and Montgomery-Asberg Depression Rating Scale (MADRS); health related quality of life: EQ-5D-5L; and functioning: Work and Social Adjustment Scale (WSAS). In-depth interviews were undertaken with 14 patients.

**Results:**

There has been high level of adherence (70%) to treatment protocol. There has been statistically significant and ‘reliable improvement’ in depression symptoms. There was statistically significant improvements in real world meaningful functioning and quality of life. Most participants described a positive impact on depressive symptoms, sleep, and functioning.

**Conclusion:**

Flow has been successfully integrated into CMHT treatment offer. It is important to offer CMHT patients an evidence-backed alternative to existing depression treatments (antiddepressant medication and talking therapies). Findings provide support for the approach of delivering together both tDCS and evidence-backed wellbeing behaviour therapy training to patients of CMHTs with experience of depression.

